# Dental caries, tobacco usage and associated risk factor of dental caries in patients visiting a government hospital in Western, Nepal

**DOI:** 10.1186/s12903-024-03997-1

**Published:** 2024-02-11

**Authors:** Krishna Subedi, Bhawana Sigdel, Purna Prasad Khanal, Deepa Sharma, Ganesh Chaudhary, Ashish Kunwar Singh, Sunil Paneru

**Affiliations:** 1 Department of Community Dentistry, Gandaki Medical College Teaching Hospital and Research Centre, Pokhara, Nepal; 2 Department of Dentistry, Pokhara Academy of Health Sciences, Pokhara, Nepal; 3 Orthodontic Unit, Dental Department, National Academy of Medical Sciences, Bir Hospital, Kathmandu, Nepal; 4 Department of Oral Medicine and Radiology, Gandaki Medical College Teaching Hospital and Research Centre, Pokhara, Nepal; 5 Dental Department, Bharatpur Hospital, Chitwan, Nepal; 6 Private dental practitioner, Pokhara, Nepal

**Keywords:** Associated risk factors, Dental caries, Nepal, Tobacco

## Abstract

**Background:**

This study was conducted to assess the prevalence of dental caries, tobacco usage, and associated risk factors for dental caries in patients who visited a government hospital in Western, Nepal.

**Methods:**

This analytical cross-sectional study was conducted from January to April 2022. Patients above 18 years visiting the dental OPD of a government hospital, and who had provided informed consent were enrolled in the study using a convenience sampling technique. As the study also involved an illiterate population, in that case, informed consent was obtained from their respective legal guardian as well. A pretested standardized, close-ended questionnaire was administered by researchers to gather information regarding the associated risk factors and oral hygiene practices. Clinical examination was done for dental caries according to the criteria by the World Health Organization (WHO) using the “DMFT” index (WHO modification 1987). Bivariate and multivariable logistic regression was done and the odds ratio and *p*-value was calculated. For all tests, statistical significance was set at *p* < 0.05.

**Results:**

A total of 219 participants completed the study with a mean age of 31.73 ± 12.46. The prevalence of dental caries and tobacco was found to be 80.36% and 5.02% respectively. Participants without health insurance had 2.35 times higher odds of dental caries (95% CI: 1.03–5.36). Not rinsing the mouth after eating sweets was associated with 3.07 times higher odds of dental caries (95% CI: 1.31–7.18). Those who hadn’t visited a dentist in the past 12 months had lower odds (0.42; 95% CI: 0.18–0.94). Eating fresh fruit daily showed statistically higher odds (2.70; 95% CI: 1.04–6.99) of dental caries. Non-tobacco users had higher odds (14.19; 2.55–78.99) of dental caries.

**Conclusion:**

Dental caries is highly prevalent, while tobacco usage is relatively low. Factors associated with dental caries included lack of health insurance coverage, consumption of fruits once daily, recent dental visits within the past year, not rinsing the mouth with water after consuming sweets, and non-tobacco users.

## Background

Dental caries is the localized destruction of susceptible dental hard tissue by acidic by-products from bacterial fermentation of dietary carbohydrates [1]. Globally, it is a major public health problem and is the most prevalent non-communicable disease (NCD). It is also the most widespread condition included in the 2015 Global Burden of Disease Study, ranking first for permanent teeth decay (affecting 2.3 billion people) and ranked 12th for primary teeth (560 million children) [2].

Caries is related to an individual’s lifestyle, and behavioral factors that are within a person’s control. These factors include inadequate oral hygiene; unhealthy dietary habits such as consuming refined carbohydrates frequently; frequent use of oral medications containing sugar; and improper feeding practices for infants. Additional factors contributing to caries risk comprise poverty, deprivation, or social status; educational attainment; dental insurance coverage; use of pit and fissure sealants; the use of orthodontic appliances; and poorly designed or ill-fitting partial dentures [3]. Nearly 60–90% of children and the vast majority of adults have dental caries, often leading to discomfort and pain [4]. Dental caries ranks among the most widespread human diseases, second only to the common cold [5]. Even though the prevalence of dental caries varies from one country to another, it remains a well-established fact that the disease exhibits a broader geographic distribution, high prevalence, and varying levels of severity [6]. Particularly, minority and economically disadvantaged populations tend to experience higher prevalence rates [7]. In Nepal, the prevalence of dental caries in 5-6-year- old and 12-13-year-old children in Nepal is reported to be 67% and 41% respectively [8].

Tobacco use is a primary cause of many oral diseases and adverse oral conditions [9]. It includes tooth loss, periodontal disease, alterations in oral soft tissues, excessive tooth wear, halitosis, discoloration of teeth, decreased taste sensation, failure of dental implants, cancer of the oropharyngeal region, and dental caries [10]. A study in England found that exposure to tobacco products for years significantly increased coronal and root caries regardless of other social and behavioral factors [11]. A systematic review and meta-analysis conducted in 2019 concluded that there is a correlation between tobacco smoking and an increased risk of dental caries [12]. In Nepal, 28.9% of adults 15–69 years of age (48.3% of men, 11.6% of women) were current users of tobacco, in any form [13].

To date, there is a lack of sufficient data regarding the prevalence and associated factors of dental caries in this particular region. Hence this study was conducted to assess the prevalence of dental caries, tobacco usage, oral hygiene practices, and associated risk factors for dental caries in patients who visited the dental department of Pokhara Academy of Health Sciences.

## Materials and methods

### Study design and participants

This analytical cross-sectional study was conducted at Pokhara Academy of Health Sciences in Kaski, Pokhara from January 2 2022 to April 29, 2022. Pokhara Academy of Health Sciences is located in the western part of Nepal, Gandaki Province, and is the largest tertiary government hospital in this province. Patients aged over 18 years visiting the dental OPD of Pokhara Academy of Health Sciences, who had provided written informed consent were enrolled in the study using a convenience sampling technique. Patients with orthodontic appliances and those with a debilitating condition that makes it difficult to examine the oral cavity were excluded.

### Ethical clearance and informed consent

Ethical clearance was obtained from the Institutional Review Committee (IRC) Pokhara Academy of Health Sciences, Kaski, Pokhara (Ref No: 30.2077/078). Written informed consent was taken from all literate participants. For the illiterate population involved in the study, informed consent was obtained from their respective legal guardians.

### Questionnaire

Researchers administered a pretested, standardized, close-ended questionnaire in the local language (Nepali) to collect information on associated risk factors and oral hygiene practices. The questionnaire includes demographic details like age, sex, education, occupation, monthly income, health insurance status, and marital status. The oral hygiene practices questions include 7 items which were: (1) How do you clean your teeth? (2) How often do you brush your teeth daily? (3) What type of toothbrush bristle do you use? (4) How often do you change your toothbrush? (5) Did you clean your teeth last time after eating sweets or chocolates? (6) Do you clean your tongue? (7) Do you use dental floss? Questionnaires related to food items include (1) Fresh fruit (2) Biscuits, cakes, cream, cakes, sweet pies, buns, etc. 3. Lemonade, Coca-Cola, or other soft drinks 4. Jam/honey 5. Chewing gum containing sugar, sweets/can, and 6. Milk/tea/coffee with sugar and their frequencies. Questions related to the utilization of dental services included: (1) visit to the dentist during the last 12 months and (2) Reason to visit the dentist. Questions assessed tobacco prevalence: Do you use tobacco? Yes/No, If yes then smoking/ smokeless tobacco. The reliability and internal consistency of the questionnaire were assessed using a test–retest approach. The questionnaire demonstrated good internal consistency with a Cronbach’s alpha value of 0.83.

### Clinical examination

Clinical examination for dental caries experience was performed according to the criteria recommended by the World Health Organization (WHO) using the “DMFT” index (WHO modification 1987) for permanent teeth [14]. A dental examination was conducted after air-drying the teeth, under artificial light, and with the aid of a mouth mirror and explorer. Six trained dentists conducted the clinical examination. Before the main study, the dentists collectively reviewed the examination methods and DMFT criteria. Inter-examiner reproducibility was evaluated using 25 randomly selected participants, who were not part of the main study.

### Sample size calculation

The sample size has been calculated using nMaster 2.0 software. This study considered (95% CI) and 7% absolute precision. As there is no national-level data regarding the prevalence of dental caries (as national-level data is present only in certain age groups), considering 50% prevalence, the sample size had been calculated as 196. Considering the 10% non-response rate, the sample size was increased to 216. However, a total of 219 cases were included in the study.

### Statistical analysis

The data were entered in Microsoft Excel 2007 and then transferred to Statistical Package for Social Sciences Version 20 software (SPSS, Inc., Chicago, IL, USA) for statistical analysis. Descriptive statistics including mean, standard deviation, and proportion were calculated, and the data were tabulated. The chi-square test and Fischer exact test were used wherever applicable. Bivariate and multivariable logistic regression analyses were conducted and the odds ratio and *p*-value were calculated. For all tests, statistical significance was set at *p* < 0.05.

## Results

The Kappa values for inter-examiner reliability for DMFT exceeded 0.87. A total of 219 completed the study with female predominance (140, 63.93%). The mean age of the participants was 31.73 ± 12.46 ranging from 18 to 77 years. As most participants refused to disclose their monthly income and occupation, these two variables were excluded from the analysis.

Approximately 30% possessed a high school education. More than half of the participants were married and enrolled in Nepal’s health insurance program (Table 1).

**Table 1:** Demographic details of the participants (*N* = 219)

Variables	Male	Female	Total
**Education**			
Illiterate	2(0.91%)	10(4.57%)	12(5.48%)
SLC and below	20(9.13%)	31(14.16%)	51(23.29%)
+ 2	30 (13.70%)	35(15.98%)	65(29.68%)
Bachelor and above	12 (5.48%)	18 (8.22%)	30(13.70%)
Not revealed	15(6.85%)	46(21.00%)	61(27.85%)
**Marital Status**			
Unmarried	36 (16.44%)	55(25.11%)	91(41.55%)
Married	43 (19.63%)	83 (37.90%)	126 (57.53%)
Separated/Widow/ Widower	-	2 (0.91%)	2 (0.91%)
**Insurance**			
Yes	45 (20.55%)	77 (35.16%)	122 (55.71%)
No	34 (15.53%)	63 (28.77%)	97 (44.29%)
**If insured, no of years**			
Less than 1 year	30 (13.70%)	39 (17.81%)	69 (31.51%)
1–2 years	9 (4.11%)	20 (9.13%)	29 (13.24%)
More than 2 years	6 (2.74%)	18 (8.22%)	24 (10.96%)

### Self-reported oral hygiene practices

About 94.06% of the respondents used toothbrushes combined with toothpaste or toothpowder for brushing their teeth. Over half of the participants brush their teeth two times or more per day. Around 43.38% of the individuals use soft toothbrushes. Almost one-third of the participants replace their toothbrushes every 3–4 months. The majority of the respondents do not rinse their mouth with water after consuming sweets or chocolates, nor do they clean their tongue. Approximately 90.41% of the participants do not use dental floss (Table 2).

**Table 2:** Self-reported oral hygiene practices of the participants (*N* = 219)

Questions	Responses	Male	Female	Total	Chi-Square	*P*-value
1. How do you clean your teeth?	**I) Toothbrush and toothpaste/toothpowder**	75 (34.25%)	131 (59.82%)	206 (94.06%)	0.169	0.77*
	**II)Toothbrush only**	4 (1.83%)	9 (4.11%)	13 (5.94%)		
2. How often do you brush your teeth daily?	**A) Once a day in the morning**	37 (16.89%)	38 (17.35%)	75 (34.25%)	NA	NA
	**B) Once a day in the evening**	5 (2.3%)	11 (5.0%)	16 (7.3%)		
	**C)Twice daily after each main meal**	35 (15.98%)	84 (38.36%)	119 (54.34%)		
	**D) more than twice daily**	1 (0.46%)	4 (1.83%)	5 (2.28%)		
	**E) Not every day**	1 (0.46%)	3 (1.37%)	4 (1.83%)		
3. What type of toothbrush bristle do you use?	A)Ultra-Soft	6(2.74%)	4(1.83%)	10(4.57%)	NA	NA
	B) Soft	35 (15.98%)	60 (27.40%)	95 (43.38%)		
	C) Medium	27 (12.33%)	59 (26.94%)	86 (39.27%)		
	D) Hard	4 (1.83%)	8 (3.65%)	12 (5.48%)		
	E) Don’t know	7 (3.20%)	9 (4.11%)	16 (7.31%)		
4. How often do you change your toothbrush?	Every month	15 (6.85%)	29 (13.24%)	44 (20.09%)	NA	NA
	B) Every 2 months	23 (10.50%)	31 (14.16%)	54 (24.66%)		
	C) Every 3–4 months	21 (9.59%)	50 (22.83%)	71 (32.42%)		
	D) Every 6 months	9 (4.11%)	13 (5.94%)	22 (10.05%)		
	E) Every year	1 (0.46%)	1 (0.46%)	2 (0.91%)		
	F) When bristles flare out	10 (4.57%)	16 (7.31%)	26 (11.87%)		
5. Did you rinse your mouth with water last time after eating sweets or chocolates?	A) Yes	31 (14.16%)	60 (27.40%)	91 (41.55%)	0.272	0.602^#^
	B) No	48 (21.92%)	80 (36.53%)	128 (58.45%)		
6. Do you clean your tongue?	A) Yes	53 (24.20%)	107 (48.86%)	160 (73.06%)	2.238	0.135^#^
	B) No	26 (11.87%)	33 (15.07%)	59 (26.94%)		
7. Do you use dental floss?	A) Yes	7 (3.20%)	14 (6.39%)	21 (9.59%)	0.076	0.783^#^
	B) No	72 (32.88%)	126 (57.53%)	198 (90.41%)		

*= Fisher exact test, ^**#**^ **= chi-square test**

### Eating/drinking patterns of the participants

More than one-third (35.62%) of the respondents included fresh fruits in their daily diet at least once daily. The majority of participants (70.32%) consume items such as biscuits, cakes, cream, sweet pies, buns, lemonade, Coca-Cola, or other soft drinks less frequently or not daily. Nearly two-thirds of the participants (64.84%) consume jam or honey, as well as chewing gum containing sugar or sweets/candy (63.47%), on a non-daily basis. Approximately 72.60% of the participants drink milk, tea, or coffee with sugar at least once a day (Table 3).

**Table 3:** Eating/drinking patterns of the participants (*N* = 219)

Items		Male	Female	Total	Chi-Square	*P*-value
Fresh fruit	Once or more than once daily	27 (12.33%)	51 (23.29%)	78 (35.62%)	NA	NA
	Not daily	52 (23.74%)	87 (39.73%)	139 (63.47%)		
	Never	0 (%)	2 (0.91%)	2 (0.91%)		
Biscuits, cakes, cream, sweet pies, buns etc.	Once or more than once daily	14 (6.39%)	23 (10.50%)	37 (16.89%)	0.243	0.886^#^
	Not daily	54 (24.66%)	100 (45.66%)	154 (70.32%)		
	Never	11 (5.02%)	17 (7.76%)	28 (12.79%)		
Lemonade, Coca Cola or other soft drinks	Once or more than once daily	5 (2.28%)	7 (3.20%)	12 (5.48%)	1.308	0.520^#^
	Not daily	54 (24.66%)	100 (45.66%)	154 (70.32%)		
	Never	20 (9.13%)	33 (15.07%)	53 (24.20%)		
Jam/honey	Once or more than once daily	4 (1.83%)	9 (4.11%)	13 (5.94%)	NA	NA
	Not daily	49 (22.37%)	93 (42.47%)	142 (64.84%)		
	Never	26 (11.87%)	38 (17.35%)	64 (29.22%)		
Chewing gum containing sugar, sweets/candy	Once or more than once daily	11 (5.02%)	19 (8.68%)	30 (13.70%)	2.966	0.227^#^
	Not daily	45 (20.55%)	94 (42.92%)	139 (63.47%)		
	Never	23 (10.50%)	27 (12.33%)	50 (22.83%)		
Milk, Tea/Coffee with sugar	Once or more than once daily	60 (27.40%)	99 (45.21%)	159 (72.60%)	0.744	0.689^#^
	Not daily	11 (5.02%)	25 (11.42%)	36 (16.44%)		
	Never	8 (3.65%)	16 (7.31%)	24 (10.96%)		

^**#**^ **= Chi-Square test**

### Visit to the dentist during the last 12 months

Nearly two-thirds (65.30%) visited a dentist during the past 12 months. The reasons for their visits included consultation/advice, pain or issues with teeth, gingiva, or mouth, and routine check-ups/follow-ups (Table 4).

**Table 4:** Visit to the dentist during the last 12 months

Variables		Male n (%)	Female n (%)	Total n (%)	Chi-square	*P*-value
Visit to the dentist during the past 12 months?	Yes	50 (22.83%)	93 (42.47%)	143 (65.30%)	0.219	0.640^#^
	No	29 (13.24%)	47 (21.46%)	34.70 (34.70%)		
Reason to visit (*n* = 143)*						
1. Consultation/advise				23 (10.5%)		
2. Pain or trouble with teeth, gums, or mouth				114 (52.1%)		
3. Routine check-up/ Follow up				34 (15.5%)		

^**#**^ = Chi-Square test, *= Multiple responses possible

### Tobacco prevalence

The overall prevalence of tobacco use was found to be 5.02% (11) [(male = 94.11% (9) female = 0.91% (2)].

### Dental caries

The prevalence of dental caries was found to be 80.36% (74.5–85.4, 95% CI). The mean DMFT was found to be 3.26 ± 3.10 (Male: 2.80 ± 2.49, Female: 3.52 ± 3.38).

### Factors associated with dental caries

Those who were not enrolled in health insurance schemes had statistically higher odds (2.35; 1.03–5.36 at 95% CI) of having dental caries. Those who had not rinsed their mouth with water after taking sweets had higher odds (3.07; 1.31–7.18 at 95% CI) of having dental caries. Those who had not visited during the last 12 months had lesser odds (0.42; 0.18–0.94 at 95% CI) of having dental caries. Eating fresh fruit at least once a day was found to be statistically associated with higher odds (2.70; 1.04–6.99 at 95% CI) of having dental caries. Participants who did not consume tobacco had higher odds (14.19; 2.55–78.99) **o**f having dental caries (Table 5).

**Table 5:** Bivariate and multivariate analysis of variables associated with dental caries among patients visiting dental OPD of government tertiary hospital in western Nepal

Variables	Dental caries		COR (95%)	AOR (95%)	*P* value
**Sex**	**Yes**	**No**			
Male	62	17	ref		
Female	114	26	1.20 (0.60 ± 2.38)	0.79 (0.34–1.82)	0.58
**Education**					
Illiterate	10	2	ref		
SLC and below	41	10	0.82(0.15–4.34)	0.45 (0.07–2.92)	0.40
Higher Secondary	52	13	0.80(0.15–4.10)	0.32 (0.05–2.13)	0.24
Bachelor and above	22	8	0.55(0.09–3.07)	0.18 (0.02–1.29)	0.08
Not revealed	51	10	1.02 (0.19–5.37)	0.40 (0.05–2.72)	0.35
**Insured**					
Yes	95	81	ref		
No	27	16	1.43 (0.72–2.85)	2.35 (1.03–5.36)	0.04
**Frequency of tooth-Brushing**					
Once or less than once a day	79	16	ref		
Twice or more than twice a day	97	27	0.72 (0.36–1.44)	0.63 (0.27–1.46)	0.28
**Rinsing mouth last time after eating sweets**					
Yes	68	23	ref		
No	108	20	1.82 (0.93–3.57)	3.07 (1.31–7.18)	0.01
**Cleaning tongue**					
Yes	132	28	ref		
No	44	15	0.62 (0.30–1.27)	0.41 (0.16–1.04)	0.06
**Use of Dental floss**					
Yes	16	5	ref		
No	160	38	1.31 (0.45–3.81)	1.45 (0.41–5.12)	0.56
**Visit to the dentist during the last 12 months**					
Yes	121	22	ref		
No	55	21	0.47 (0.24–0.93)	0.42 (0.18–0.94)	0.03
**Fresh fruit**					
Not daily/Never	107	34	ref		
Once or more than once daily	69	9	2.43 (1.10–5.39)	2.70 (1.04–6.99)	0.04
**Biscuits, cakes, cream, sweet pies, buns etc.**					
Never	21	7	ref	-	-
Not daily	124	30	1.37 (0.53–3.54)	0.51 (0.12–2.20)	0.37
Once or more than once daily	31	6	1.72 (0.50–5.85)	0.95 (0.16–5.46)	0.95
**Lemonade, Coca Cola or other soft drinks**					
Never	42	11	ref	-	-
Not daily	124	30	1.08 (0.49–2.34)	1.09 (0.40–2.95)	0.86
Once or more than once daily	10	2	1.31 (0.25–6.86)	0.44 (0.06–3.14)	0.41
**Jam/honey**					
Never	49	15	ref	-	-
Not daily	116	26	1.36 (0.66–2.80)	1.78 (0.73–4.30)	0.19
Once or more than once daily	11	2	1.68 (0.33–8.45)	1.13 (0.19–6.78)	0.88
**Chewing gum containing sugar, sweets/candy**					
Never	38	12	ref	-	-
Not daily	112	27	1.31 (0.60–2.83)	1.22 (0.37-4.00)	0.73
Once or more than once daily	26	4	2.05 (0.59–7.06)	1.65 (0.32–8.53)	0.54
**Milk, Tea/Coffee with sugar**					
Never	19	5	ref	-	-
Not daily	26	10	0.68 (0.20–2.33)	0.96 (0.23–3.88)	0.95
Once or more than once daily	131	28	1.23 (0.42–3.57)	1.53 (0.44–5.23)	0.49
**Tobacco use**					
Yes	6	5	ref	-	-
No	170	38	3.72 (1.08–12.85)	14.19 (2.55–78.99)	0.002

## Discussion

The prevalence of dental caries and tobacco use was found to be 80.36% and 5.02% respectively. In this study, dental caries was associated with several factors, including lack of health insurance coverage, daily consumption of fruits, recent dental visits within the last 12 months, not rinsing the mouth with water after consuming sweets, and abstaining from tobacco usage. Almost all participants used toothbrushes combined with toothpaste or toothpowder for brushing their teeth. Over half of the participants brush their teeth two times or more per day. Most respondents didn’t rinse their mouth with water after eating sweets or chocolates, and they also neglected to clean their tongue or use dental floss.

The prevalence of dental caries was found to be 80.36% which is almost similar to the studies done by Karki et al. [15] (79.26%), Bhagat et al. [16] (83.0%) but higher compared to studies done by Khapung et al. [17] (57.74%) and Singh et al. [18] (57.5%). This discrepancy may be due to the hospital setting of this study, while data collection in the other two studies was at the community level. This study showed that almost 95% use toothbrushes and toothpaste/toothpowder for cleaning and 58.62% brushed their teeth twice or more than twice daily, higher than the study done by Thapa et al. [19] where 88.2% used toothbrushes for cleaning and only 10% brush twice daily. This may be attributed to increased awareness over time and the easy availability of toothbrushes.

Almost 10% of the study population uses dental floss which seems to be higher as compared to a study done by Erchick et al. [20] on pregnant women in Nepal (less than 1% use dental floss). Additionally, 41.55% of the participants rinse their mouth with water last time after eating sweets or chocolates which was almost similar to a study done by Gai et al. [21] in school children where 40.1% rinse their mouth after food.

1. 73.06% clean their tongue which is higher compared to a study done by Tadin et al. [22] where almost 60.0% clean their tongue daily.
2. 65.30% of participants visited the dentist during the last 12 months with the main reason being pain or trouble with teeth, gums, or mouth. This is almost similar to a study done in the United States [23] where 69.6% visited the dentist within the last 12 months.

Overall the prevalence of tobacco was found to be 5.02% (11) which is very low compared to tobacco prevalence in Nepal, which is 28.9% [24].

Those who were not insured had higher odds of dental caries compared to those with insurance. This could be because individuals with insurance may have visited the hospital and received treatment for dental caries.

Those who had visited a dentist during the last 12 months had higher odds of having dental caries which is almost similar to the study done by Ndagire et al. [25] where adolescents who had visited a dentist previously had 1.4 times the prevalence of developing dental caries than those who never visited a dentist. This may be because they experienced pain or other symptoms, leading them to visit dentists more frequently.

The odds of experiencing dental caries was found to be higher in those who consumed fruits once or more than once daily, which is similar to a study done by Arora et al. [26], where each serving of fruit increased the odds of experiencing caries by 52%. The association between dental caries and fruit consumption may be attributed to the acid present in fruits. However, further longitudinal studies are required to confirm this association.

The odds of experiencing dental caries was found to be higher in those who did not rinse their mouth water after eating sweets which is similar to a study done by Shitie et al. [27] Not rinsing the mouth after consuming sweets may cause the accumulation of sweets between the teeth, leading to acid production and potential dental caries.

Higher odds of experiencing dental caries were in those who were not chewing tobacco. This contrasts with the finding of a systemic review [12] that suggested an association between smoking and dental caries. This variation may be due to a small sample size, as only 11 (5.02%) participants were using tobacco. If a larger number of tobacco users were included, the association might change.

### Limitations of the study

Due to the cross-sectional design of the study, no causal associations could be established. Additionally, the dietary history relied on recollections from the past 24 h/weeks, introducing potential recall bias as participants may not accurately remember their actual diet frequency. Regarding tobacco use, participants might not have provided precise information due to social desirability bias. Additionally, the association between tobacco use and dental caries may not be reliable due to the small number of tobacco users in the study. Furthermore, since the data was collected from a single center, it cannot be generalized to the entire country.

## Conclusion

The prevalence of dental caries is very high and tobacco usage is low. Factors such as lack of health insurance coverage, regular consumption of fruits once or more daily, recent dental visits within the last 12 months, not rinsing the mouth with water after consuming sweets, and abstaining from tobacco usage were associated with dental caries.

## Data Availability

Data will be made available upon reasonable request to the corresponding author (Krishna Subedi).

## References

[1] Longbottom C, Huysmans MC, Pitts NB, Fontana M. Glossary of key terms. Detection, assessment, diagnosis and monitoring of caries. 2009;21:209–16. 10.1159/000224225 19494688

[2] World Health Organization. Sugars and dental caries. https://apps.who.int/iris/bitstream/handle/10665/259413/WHO-NMH-NHD-17.12-eng.pdf;jsessionid=C000F91A43E3610BCCB0D03F526C6955?sequence=1. Accessed 7th September 2020.

[3] Selwitz RH, Ismail AI, Pitts NB (2007). Dental caries. Lancet.

[4] World Health Organization. Oral health. What is the burden of oral disease? https://www.who.int/oral_health/disease_burden/global/en/. Accessed 27th September 2020.

[5] Islam B, Khan SN, Khan AU (2007). Dental caries: from infection to prevention. Med Sci Monit.

[6] Olabisi A, Udo U, Adeniyi A, Bashiru B, Ehimen U, Gbenga O (2015). Prevalence of dental caries and oral hygiene status of a screened population in Port Harcourt, Rivers State, Nigeria. J Int Soc Prev Community Dent.

[7] Teshome A, Andualem G, Derese K (2020). Dental Caries and Associated factors among patients attending the University of Gondar Comprehensive Hospital Dental Clinic, North West Ethiopia: A Hospital-based cross-sectional study. Clin Cosmet Investig Dent.

[8] National oral health policy. 2004. https://www.mohp.gov.np/downloads/National%20Oral%20Health%20Policy.pdf. Accessed 27th September 2020.:8.

[9] World Health Organization. Oral health. Global facts on tobacco or oral health. https://www.who.int/oral_health/publications/orh_factsheet_wntd.pdf?ua=1. Accessed 27th September 2020.

[10] Reibel J (2003). Tobacco and oral diseases. Med Princ Pract.

[11] Jette AM, Feldman HA, Tennstedt SL (1993). Tobacco use: a modifiable risk factor for dental disease among the elderly. Am J Public Health.

[12] Jiang X, Jiang X, Wang Y, Huang R. Correlation between tobacco smoking and dental caries: a systematic review and meta-analysis. Tob Induc Dis. 2019;17. 10.18332/tid/106117 PMC6662788 31516477

[13] Nepal STEPSS. 2019 Tobacco Fact Sheet. http://nhrc.gov.np/wp-content/uploads/2019/11/Tobacco-Fact-Sheet-1.pdf. Accessed 28th September 2020.

[14] World Health Organization. Oral health surveys-Basic methods, Geneva, 5th edition. 2013:1–137. Available from: https://apps.who.int/iris/bitstream/handle/10665/97035/9789241548649_eng.pdf?sequence=1&isAllowed=y. Accessed 2020 Aug 18.

[15] Karki B, Kunwar S, Gaire G, Magar KR, Bhusal L, Giri P (2023). Dental Caries among patients visiting the Dental Outpatient Department in a Tertiary Care Centre: a descriptive cross-sectional study. J Nepal Med Assoc.

[16] Bhagat T, Rao A, Shenoy R (2013). Assessment of oral Health Status of 35–44 and 65–74 Year old adults in Bairawa, Saptari, Nepal. Indian J Contemp Dent.

[17] Khapung A, Shrestha S (2022). Dental Caries among Adult Population of a municipality: a descriptive cross-sectional study. JNMA J Nepal Med Assoc.

[18] Singh A, Shrestha A, Bhagat TK, Baral DD (2020). Assessment of oral health status and treatment needs among people of Foklyan area, Dharan, Nepal. BMC Oral Health.

[19] Thapa P, Aryal KK, Mehata S, Vaidya A, Jha BK, Dhimal M (2016). Oral hygiene practices and their socio-demographic correlates among Nepalese adult: evidence from non communicable diseases risk factors STEPS survey Nepal 2013. BMC Oral Health.

[20] Erchick DJ, Rai B, Agrawal NK, Khatry SK, Katz J, LeClerq SC (2019). Oral hygiene, prevalence of gingivitis, and associated risk factors among pregnant women in Sarlahi District, Nepal. BMC Oral Health.

[21] Pai NG, Acharya S, Vaghela J, Mankar S (2018). Prevalence and risk factors of dental caries among school children from a low socio economic locality in Mumbai, India. Int J Appl Dent Sci.

[22] Tadin A, Poljak Guberina R, Domazet J, Gavic L (2022). Oral Hygiene practices and oral health knowledge among students in Split, Croatia. Healthcare.

[23] Lutfiyya MN, Gross AJ, Soffe B, Lipsky MS (2019). Dental care utilization: examining the associations between health services deficits and not having a dental visit in past 12 months. BMC Public Health.

[24] Nepal Demographic health survey. 2022. Available from httpsdhsprogram.compubspdfFR379FR379.pdf.pdf. Accessed 24th July 2023.

[25] Ndagire B, Kutesa A, Ssenyonga R, Kiiza HM, Nakanjako D, Rwenyonyi CM (2020). Prevalence, severity and factors associated with dental caries among school adolescents in Uganda: a cross-sectional study. Braz Dent J.

[26] Arora A, Evans RW (2012). Is the consumption of fruit cariogenic? Cariogenicity of fruit. J Investig Clin Dent.

[27] Shitie A, Addis R, Tilahun A, Negash W (2021). Prevalence of Dental Caries and its Associated factors among primary School children in Ethiopia. Int J Dent.

